# Gastrointestinal nematodes in German outdoor-reared pigs based on faecal egg count and next-generation sequencing nemabiome data

**DOI:** 10.1186/s40813-024-00384-8

**Published:** 2024-09-12

**Authors:** Hannah R. M. Fischer, Jürgen Krücken, Stefan Fiedler, Veronica Duckwitz, Hendrik Nienhoff, Stephan Steuber, Ricarda Daher, Georg von Samson-Himmelstjerna

**Affiliations:** 1 Institute for Parasitology and Tropical Veterinary Medicine, Freie Universität Berlin, Robert-von-Ostertag-Str. 7, 14163 Berlin, Germany; 2 Veterinary Centre for Resistance Research (TZR), Freie Universität Berlin, Robert-von-Ostertag-Str. 8, 14163 Berlin, Germany; 3 Federal Office of Consumer Protection and Food Safety (BVL), Gerichtstr. 49, 13347 Berlin, Germany; 4 Institute of Veterinary Epidemiology and Biostatistics, Freie Universität Berlin, Königsweg 67, 14163 Berlin, Germany; 5 Institute for Animal Health (Lufa-Nord-West), Ammerländer Heerstr. 123, 26129 Oldenburg, Germany

**Keywords:** Pig, Nematode, FLOTAC, ITS-2, Deep amplicon sequencing, Nemabiome

## Abstract

**Background:**

There is a higher risk for nematode infections associated with outdoor-reared pigs. Next to *Ascaris suum*, *Oesophagostomum dentatum* and *Trichuris suis*, there is the potential of infections with other nodular worm species, *Hyostrongylus rubidus, Stongyloides ransomi* and *Metastrongylus* spp. lungworms. Next-generation sequencing methods describing the nemabiome have not yet been established for porcine nematodes.

**Methods:**

FLOTAC was used for faecal egg counts of porcine gastrointestinal nematodes and lungworms in piglets, fatteners and adults individually. A nemabiome analyses based on ITS-2 gene region metabarcoding was used to differentiate strongyle species. Additionally, questionnaire data was analysed using mixed-effect regression to identify potential risk factors associated with parasite occurrences and egg shedding intensity.

**Results:**

On 15 of 17 farms nematode eggs were detected. *Ascaris suum*, strongyles and *T. suis* were detected on 82%, 70% and 35% of the 17 farms, respectively. Lungworms were detected on one out of four farms with access to pasture. *Strongyloides ransomi* was not detected. 32% (CI 28–36%), 27% (24–31%), 5% (4–7%) and 3% (0.9–8%) of the samples where tested positive for strongyles, *A.* *suum*, *T. suis* and lungworms, respectively. The nemabiome analysis revealed three different strongyle species, with *O. dentatum* being the most common (mean 93.9%), followed by *O.* *quadrispinulatum* (5.9%) and the hookworm *Globocephalus urosubulatus* (0.1%). The bivariate and multivariate risk factor analyses showed among others that cleaning once a week compared to twice a week increased the odds significantly for being infected with *A.* *suum* (OR 78.60) and strongyles (2077.59). Access to pasture was associated with higher odds for *A. suum* (43.83) and strongyles (14.21). Compared to shallow litter systems, deep litter and free range systems resulted in significant higher odds for strongyles (85.74, 215.59, respectively) and *T. suis* (200.33, 623.08).

**Conclusions:**

Infections with *A.* *suum*, *O.* dentatum*, O.* *quadrispinulatum, T. suis*, *Metastrongylus* spp. and *G.* *urosubulatus* are present in German outdoor-reared pigs. This is the first report of *G.* *urosubulatus* in domestic pigs in Europe. Metabarcoding based on the ITS-2 region is a suitable tool to analyse the porcine nemabiome. Furthermore, management practices have the potential of reducing the risk of parasite infections.

**Supplementary Information:**

The online version contains supplementary material available at 10.1186/s40813-024-00384-8.

## Background

Germany is the second largest pig producer in Europe [1]. Since animal welfare is an increasingly important consumer decision criterion, animal access to outdoor areas is increasingly in demand. This becomes apparent in the proportion of pigs reared as EU-certified organic pigs which increased between 2010 and 2020 by about 36% [2]. Overall, alternative farming of pigs can be divided into two groups in Germany: farms with non-slatted concrete outdoor areas (COA, which do not necessarily have to be certified organic farms) and farms with pasture access. Noteworthy, compared to conventional indoor pig production, alternative pig production is, as a result of favourable conditions for helminth transmission [3–5], associated with higher nematode prevalences [6, 7]. Nematode infection in pigs often do not lead to clinical disease [8, 9], but especially with high worm burden they may impact the performance of the pigs [10–20] and therefore, be of economic importance.

To improve the control of porcine nematode infections in alternative farm systems it is of interest to know the potentially underlying factors for the distribution and occurrence of these parasites. Studies investigating parasite distributions in farms with outdoor access have previously been performed in 2002 in Denmark [3], in 2005 in the Netherlands [6], in 2017 in Poland [7], in 2020 in Sweden [21] and in 2023 in the USA [22]. So far, no comparable studies have been carried out in Germany and there is a lack of up-to-date information on the occurrence of the various porcine nematode species in German outdoor reared pigs.

Parasite control in a pig herd may be successfully accomplished by a combination of the use of anthelmintic drugs and diverse hygiene and biosecurity measures [23]. However, the use of anthelmintic drugs and its efficacy is restricted by several factors. First, the purely meta-/prophylactic use of anthelmintic drugs is forbidden in the EU organic regulations (EU Council Regulation (EC) 834/2007 and Commission Regulation 889/2008). Secondly, previous studies had difficulties correlating anthelmintic treatment with helminth prevalence [23–26], implying that reinfection is common and treatment effects are only transitory if shed eggs and infective larvae remain viable in the environment. Finally, anthelmintic drug resistance is a significant and complex issue in ruminant livestock [27] and has also been reported for *Oesophagostomum* spp. in pigs for different active components, such as ivermectin, pyrantel, levamisole and benzimidazoles [28–32]. Overall, this emphasizes the importance of alternative control mechanisms. Therefore, it is important to conduct risk factor analyses in order to reveal parameters affecting the parasite occurrence.

The main prevalent nematode species in pig production in temperate climates are *Ascaris suum*, *Oesophagostomum* spp. and *Trichuris suis* [8]. In free range pigs there is the additional potential of lungworm, i.e. *Metastrongylus* spp. infections, due to the access to the intermediate host, the earthworm. Furthermore, compared to intensive indoor reared pigs, outdoor reared pigs may have a higher risk of getting in contact with wild boars or their shed helminth eggs. Traditionally the species differentiation of strongyles is carried out by cultivation of L3 and the morphological differentiation of larvae. However, this method is time consuming, relatively labour intensive and may result in under diagnosing low level infections since typically only 100 L3 are differentiated per sample. As an alternative method, Avramenko et al. [33] introduced the nemabiome metabarcoding based on the ITS-2 rRNA gene region as a suitable tool for species differentiation in ruminants. This approach subsequently has been used for different studies investigating intestinal parasites in cattle [34–36], sheep [37, 38], goats [39], roe deer [37], horses [40, 41] and primates [42]. However, it has not yet been applied to characterize the nematode communities in pigs.

The present study aimed to assess the occurrence of parasitic nematodes in German alternatively reared pigs in vivo. Moreover, a risk factor analysis based on a questionnaire was performed, correlating management practices with parasite occurrence and egg shedding intensity, in order to disclose potential control parameters. Finally, nemabiome metabarcoding was implemented for the first time to characterize the nematode community in pigs.

## Materials and methods

### Faecal samples

Faecal samples (n = 607) were collected between February 2022 and March 2023 from voluntarily participating German alternative pig rearing farms with outdoor areas. Only farms that employed at least a non-slatted concrete outdoor area were included. In order to compare different farm systems, it was also aimed to analyse faecal samples from animals with access to pasture. Where possible, individual rectal faecal samples from up to 30 piglets (2–3 weeks after weaning, about 10 weeks old), up to 30 fatteners (24–26 weeks old; approximately 4 weeks prior to slaughter) and up to 20 adult swine, mainly sows, were collected per farm. Only animals were included that had not been treated with anthelmintics for at least 6 weeks prior to sampling. The faeces were collected rectally using examination gloves, then sealed with a knot and immediately cooled in a transportable cooler (4–8 °C). The samples were kept cool (4–8 °C) for up to 3 days post sampling until further processing in the laboratory.

### Questionnaire

For statistical analyses, each participating farmer was interviewed about the management system on the farm using a questionnaire. Data on four main subject areas, such as general information, animal husbandry, treatment and hygiene procedures were collected. For detailed information, see the complete, translated questionnaire in Additional File 1: Text S1.

### Faecal egg count via FLOTAC

All samples were analysed individually using the FLOTAC technique as previously described [43] with saturated sodium chloride (specific gravity 1.20) and 10 g of faeces weighed on a scale. The number of all occurring eggs per nematode egg type (strongyle, ascarid, *Trichuris suis, Strongyloides ransomi* and *Metastrongylus* spp. eggs) was counted in both counting chambers of the FLOTAC device resulting in a multiplication factor of 1.

### Egg isolation

The faeces of fatteners were pooled per farm. For breeding farms, faeces of adults and piglets were pooled together. The pooled faeces were subsequently homogenized in tap water and filtered through a kitchen sieve (> 200 µm mesh size). The collected suspension was then poured through a 25 µm sieve. The eggs collected on top of the sieve were thoroughly rinsed with tap water and then collected. After centrifuging (1000 × g, 5 min), the supernatant was discarded and the remaining pellet containing the eggs was resuspended with saturated sodium chloride solution. After another centrifugation (1000 × g, 5 min), the eggs floating in the suspension were rinsed on a 25 µM sieve to remove excess sodium chloride and were further separated from faecal particles using a sugar step gradient. For processing pig nematode eggs four different sugar gradient solutions were used to obtain the best results: A sugar solution obtained by solving 60 g sucrose in 40 mL distilled water was designated 100% sugar solution. Based on this, the 10%, 25%, 40% and 60% sugar solutions were obtained by dilution with tap water. Each sugar solution was stained individually with food colour to facilitate layering and detection of the eggs. Step gradients were prepared by adding 10 ml of each solution into a 50 mL centrifugation tube starting with the solution with the lowest density and stepwise addition of the other underlayers. Up to 10 mL of the egg suspension were added on the top of the sugar gradient and then centrifuged (1000 × g, 5 min, break deactivated). The gastrointestinal strongyle eggs were found between the 25% and 10% layers. The eggs of *A.* *suum* have a higher density and were usually found between the 60% and 40% layers. The purified eggs were frozen at −20 °C in 300 µL aliquots (range 45–9000 strongyle eggs) until further processing.

### Genomic DNA extraction

For extraction of genomic DNA from nematodes eggs, the NucleoSpin® Soil (Macherey-Nagel®, Düren, Germany) was used following the producer’s protocol. Samples (300 µL) were lysed using 700 µL of the SL1 lysis buffer and mechanical beat beating in the kit’s lysis tubes. DNA was eluted with 50 µL DEPC-treated water and frozen at −20 °C until further processing.

### Nemabiome analyses: Internal-transcribed-spacer-2 PCRs and deep amplicon sequencing

Based on ITS-2 amplicons generated from strongylid egg DNA harvested from pooled faecal samples, a deep amplicon sequencing was performed as described previously for ruminants [33, 34]. The PCR amplification was carried out with modified primers NC1 and NC2 [44] with Illumina Adapter sequencing tag, as previously described [33, 45]. Overall, four forward (Illumina-NC1-0N/3N) and four reverse (Illumina-NC2-0N/3N) primers (Integrated DNA Technologies, Inc., Coralville, Iowa, USA; standard desalting) with 0 to 3 random nucleotides (N) added between the NC1/NC2 primer sequences and the Illumina adapter to avoid fluorescence signal saturation during Illumina sequencing [33], were mixed in equal measures. The PCR reaction contained 0.3 µM of each NC1 and NC2 primer mix and 8 µl template DNA in 50 µL 1× Kapa HiFi HotStart ReadyMix (Roche Molecular Systems, Peasanton, CA, USA). Cycling conditions were: 95 °C for 2 min, followed by 30 cycles at 98 °C for 20 s, 62 °C for 15 s and 72 °C for 15 s, and finally an extension at 72 °C for 2 min. PCR products were purified with AMPure XP Magnetic Beads (Beckman Coulter GmBH, Krefeld, Germany) following the manufacturer’s protocol with a 1:1 sample/bead ratio and eluted in 40 µL 10 mM Tris-HCl (pH 8.0). The concentration of each purified amplicon was determined using the Qubit dsDNA HS Assay Kit (Thermo Fisher Scientific, Darmstadt, Germany) on a Qubit 4 Fluorometer (Thermo Fisher Scientific, Darmstadt, Germany). Illumina indices and P5/P7 sequencing tags were then added to the ITS-2 amplicons using limited cycle PCR amplification, with the following PCR reactions: 10-20 ng purified first round PCR product and 2.5 µL IDT for Illumina DNA/RNA dual index primers (Illumina, San Diego, California USA) in 25 µl of Kapa HiFi Ready Mix. The thermocycling parameters were: 98 °C for 45 s, followed by 7 cycles at 98 °C for 20 s, 63 °C for 20 s and 72 °C for 120 s, followed by a final extension at 72 °C for 120 s. The indexed PCR products were then cleaned again using AMPure XP Magnetic Beads according to the manufacturer’s protocol (1:1 sample/bead ratio; elution volume 25 µL 10 mM Tris-HCl buffer pH 8.0). The DNA concentration was again quantified using the Qubit dsDNA HS Assay Kit. The final libraries were then diluted to 4 nM in 10 mM Tris-HCl buffer (pH 8.0) and pooled according to the Illumina protocol. The prepared pooled library was run on a MiSeq benchtop sequencer (V3, 2 × 300 bp, Illumina). It was aimed to generate at least 20,000 paired end reads for each sample.

### Nemabiome analyses: species assignment via dada2 pipeline

Species assignment was performed using the analysis pipeline described in detail on the nemabiome web page [46]. Sequence reads were automatically de-multiplexed by the MiSeq software. First, primer and adapter sequences were removed using cutadapt v3.5-1 [47]. The following steps were performed with the R package dada2 v1.28.0 [48]. Reads were quality filtered with maximum expected errors of 2 for the forward sequence and five for the reverse sequence. Reads were then truncated to a quality score of maximum 2 expected errors per read. Next, dada2 was taught an error profile using the actual sequence data set to be analysed. This generated error profile was used for denoising the sequences with the dada2 algorithm. Denoised paired-end reads were subsequently merged to reconstruct the full amplicon sequence. After removal of chimeras with dada2, taxonomic assignment was performed using IdTaxa from the R package DECIPHER v2.28.0 [49, 50] and the nemabiome ITS-2 database v1.5.0 [51] from the aforementioned nemabiome web page. The settings for IdTaxa were chosen as follows: the threshold was set at 60 and the bootstrap repeats at 100. BLASTN search [52] against the GenBank database was used for amplicon sequence variants (ASVs) which could not be positively identified via IdTaxa. Non-strongyle ASVs were excluded from further analyses.

### Phylogenetic analysis *Oesophagostomum* spp.

To further characterize *Oesophagostomum* spp. sequences that could not be assigned to a particular species, a phylogenetic tree based on ITS-2 sequences from GenBank and the *Oesophagostomum* ASVs from deep amplicon sequencing was generated. *Chabertia ovina* ITS-2 sequence was used as outgroup. Sequences were truncated to the ITS-2 region and an alignment was created with MAFFT [53] using the Q-INS-i option and the “leave gappy regions” option. A maximum-likelihood phylogenetic tree was calculated with the IQ-TREE web server using version 1.6.12 for Linux [54, 55]. For the model selection, ModelFinder [56] was used and „FreeRate heterogeneity“ was included in the model search. Branch support was calculated by means of ultrafast bootstraping (UFBoot) [57] and the Shimodaira-Hasegawa (SH)-like approximate likelihood ratio test (aLRT) [58]. The generated phylogenetic tree in Newick format was visualized using FigTree v1.4.4 [59].

### Statistical analyses

Statistical analyses were calculated in either R v4.2.1 or GraphPad Prism v5.02 for Windows (GraphPad Software, San Diego, California USA, www.graphpad.com). For nematode frequency calculations with infinite population sizes the binom.wilson function from the R package epitools v0.5-10.1 [60] was used for proportion and confidence interval (CI) calculations. At farm level with specific/definite population sizes the CONF.prop function from the R package stat.extend v0.2.1 [61] was used to calculate CI for nematode prevalence. Risk factor analysis using generalized linear mixed models (GLMMs) was performed with the function glmmTMB from the R package glmmTMB v.1.1.7 [62] in bivariate and multivariate models for *A. suum*, strongyles and *T. suis* independently. The farm ID was continuously factored in as random effect variable. Binomial analysis concerning the infection status, was performed considering animals with a faecal egg count per gram (EPG) of ≥ 1 as positive and animals with an EPG of 0 as negative. For analysing the risk factors for the egg shedding intensity, only the EPGs of positive animals were taken into account and the family „truncated_nbinom2“ was used for the calculations via the glmmTMB function. As a criterion for model selection and to optimize the model fit both the R package DHARMa v0.4.6 [63] and the Akaike information criterion (AIC) were used for choosing the best fit for the multivariate models. To identify significant differences between prevalence and EPG in different age groups a Kruskal-Wallis followed by Dunn’s post hoc test via GraphPad Prism was applied. For correlation analysis a Spearman’s rank correlation coefficient was calculated with the GraphPad Prism software.

## Results

### Included farms

**Fig. 1:**
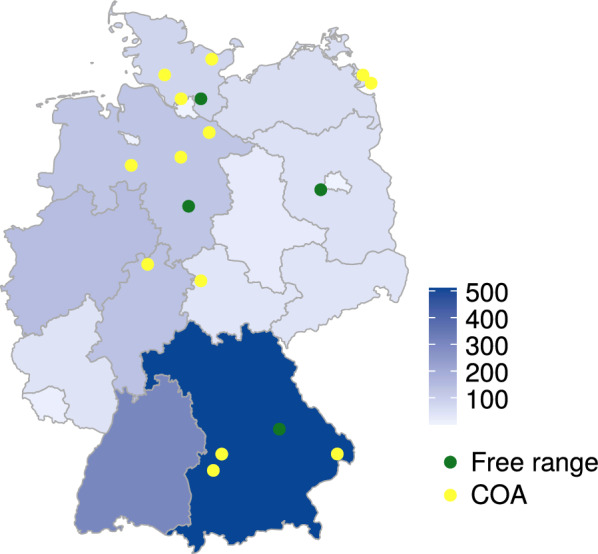
Number of organic farms in German federal states and the localization of the participating farms. The colour of each federal state indicates the total number of registered organic farms per state, as indicated in the legend. The localization of the participating farms is shown with green circles for farms where at least one age group of sampled animals had access to pasture and yellow circles for farms with non-slatted concrete outdoor areas (COA). The map was generated using the R package choroplethrAdmin1 v1.1.1 and the data originates from the 2020 agricultural census of the Federal Statistical Office of Germany

In total, seventeen farms in seven different federal states of Germany (Fig. 1) participated in the study. Five farms had free-range areas for different age-groups. Of these, only four allowed sampling animals which had access to pasture (farms 4, 10, 11 and 14). Sixteen farms provided non-slatted concrete outdoor areas (COA) for the animals. Only farm 14 was an exclusively free-range farm. Farm 2 was visited twice, as indicated in the numbering (2.1, 2.2). Farm 13 had two locations, of which one was the fattening farm (13a) and one the breeding farm (13b). An overview of the included farms, number of tested animals and animal husbandry form is provided in Table 1.

**Table 1:** Overview of farms and no. of tested animals in the study

Farm	Total no. animals^a^	Sows tested (total no.)	Boars tested (total no.)	Fatteners tested (total no.)	Piglets tested (total no.)	Animal husbandry	
						COA^b^	Free-range
1	349	8 (32)	1 (1)	21 (180)	19 (88)	S^c^, B^d^, F^e^, P^f^	–
2.1^g^	150	–	–	30 (150)	–	F	–
‍2.2^g^	140	–	–	27 (140)	–	F	–
‍3	180	12 (35)	0 (3)	0 (15)	30 (75)	S, B, P	F
‍4	289	13 (45)	–	31 (96)	28 (79)	S, F	S, P
‍5	150	–	–	29 (150)	–	F	–
‍6	450	7 (75)	1 (2)	0 (100)	8 (250)	S, B, F, P	–
‍7	172	–	–	30 (172)	–	F	–
‍8	37	–	–	19 (37)	–	F	–
‍9	95	6 (8)	–	29 (58)	15 (19)	S, F, P	–
‍10	173	12 (16)	1 (1)	0 (7)	30 (149)	S, B, F, P	S
‍11	94	4 (4)	0 (1)	0 (42)	10 (25)	S, P	S, B, F
‍12	323	13 (42)	0 (1)	0 (9)	27 (98)	S, B, F, P	–
‍13a^h^	1500	–	–	21 (1500)	–	F	–
‍13b^h^	1502	16 (300)	0 (2)	–	25 (600)	S, B, P	–
‍14	120	0 (9)	0 (1)	0 (70)	21 (25)	S, P	S, B, F
‍15	940	–	–	30 (940)	–	F	–
‍16	540	–	–	29 (540)	–	F	–

^a^Total no. animals also includes other age groups that were not sampled (e.g. suckling piglets); ^b^concrete outdoor area; ^c^sows; ^d^boars; ^e^fatteners; ^f^piglets; ^g^2.1/2.2, first and second visit on farm 2, respectively; ^h^13a/b, fattening/breeding unit of one farm with two locations

### Frequency of positive animals and farms

The FLOTAC device with a multiplication factor of 1 was used to count the EPG of strongyle, ascarid, *T.* *suis* and *Metastrongylus* spp. eggs. There was no detection of *S.* *ransomi* eggs. In the following text, two different terms for the occurrence of nematode species are used. At farm level, the term prevalence will be used, but at the level of Germany the term frequency was chosen because the study population is not representative for German outdoor-reared pigs.

Overall, *A.* *suum* was detected on 14 (82%), strongyle eggs on 12 (70%) and *T.* *suis* on six (35%) of 17 farms. Lungworms were detected on one out of four farms with access to pasture. On two farms (12, 16) no nematode eggs were detected.

Of the total samples tested, the overall frequency of strongyles (n = 577), ascarids (n = 607), whipworms (n = 607) and lungworms (n = 108) across all age groups was 32% (95% CI 28–36%), 27% (24–31%), 5% (4–7%) and 3% (0.9–8%), respectively (see Table 2 for details and reasons why the number of animals varies between parasite groups). The egg shedding of strongyles was significantly higher in adults (mean EPG = 448) than in piglets (*p* < 0.001) and fatteners (*p* < 0.001), of ascarids higher in fatteners (EPG = 150) than in sows (*p* < 0.001) and piglets (*p* < 0.01) and, of whipworms higher in fatteners (EPG = 2) than in sows (*p* < 0.01) and piglets (*p* < 0.001). The mean EPG of lungworms in piglets was very low with only 0.2 EPG, other age groups were all tested negative. However, it has to be taken into account that on the farm tested positive for lungworms (14), due to the lacking accessibility of the sows and fatteners matching the study criteria, only piglets were sampled. For strongyle egg shedding, there was a significant positive correlation between egg shedding of sows and piglets from the same farm (Spearman’s rank correlation coefficient *p* = 0.84, *p* < 0.01).

**Table 2:** Frequency of strongylid nematodes, *A. suum*, *T. suis* and *Metastrongylus* spp. based on faecal egg counts using the FLOTAC device and mean, median and range of the EPG

	Age group (no. of samples)	% Frequency (95% CI)	EPG		
			Mean (SD)	Median (Q1, Q3)	Range
**Strongyles**	Piglets (217)	28 (22–34)	167 (739.5)	0 (0, 2)	0–5871
	Fatteners (266^a^)	28 (24–34)	28 (92)	0 (0, 4)	0–806
	Adults (94)	44 (34–54)	448 (937)	0 (0, 451)	0–4520
	**Total (577**^**a**^**)**	**32 (28–36)**	**148 (610)**	**0 (0, 6)**	**0–5871**
***Ascaris suum***	Piglets (217)	23 (18–29)	67 (223)	0 (0, 0)	0–1342
	Fatteners (296)	37 (31–42)	150 (637)	0 (0, 4)	0–6496
	Adults (94)	6 (3–13)	3 (16)	0 (0, 0)	0–128
	**Total (607)**	**27 (24–31)**	**98 (467)**	**0 (0, 1)**	**0–6496**
***Trichuris suis***	Piglets (217)	1 (0.5–4)	0.03 (0.3)	0 (0, 0)	0–4
	Fatteners (296)	9 (7–13)	2 (12)	0 (0, 0)	0–151
	Adults (94)	1 (0.2–6)	0.03 (0.3)	0 (0, 0)	0-3
	**Total (607)**	**5 (4–7)**	**1 (8)**	**0 (0, 0)**	**0-151**
***Metastrongylus* spp.**	Piglets (49^b^)	6 (2–17)	0.2 (0.8)	0 (0, 0)	0–5
	Fatteners (29^b^)	0 (0–12)	0 (0)	0 (0, 0)	0–0
	Adults (30^b^)	0 (0–11)	0 (0)	0 (0, 0)	0–0
	**Total (108**^**b**^**)**	**3 (0.9–8)**	**0.07 (0.52)**	**0 (0, 0)**	**0–5**

In order to destinguish the numbers concerning all three age groups (Total) from single age groups (piglets, fatteners, adults) for each parasite, it was chosen to highlight them in bold

EPG, egg per gram; CI, confidence interval; SD, standard deviation; Q1, first quartile; Q3, third quartile

^a^Strongyle egg count data from 30 samples from farm 2.1 are not reliable and were therefore excluded from further analysis

^b^Only samples of animals with access to pasture and therefore, with the potential for lungworm infections were included

**Fig. 2:**
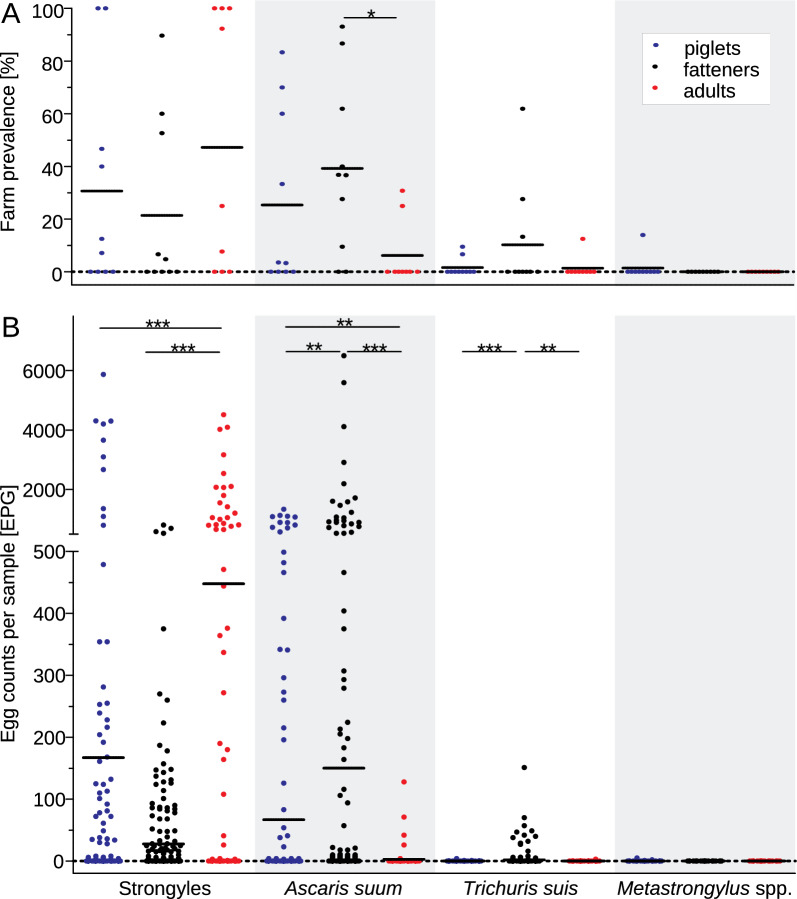
Farm prevalence in percent **A** and individual egg counts in egg per gram faeces (EPG) **B** of strongyles, *Ascaris suum*, *Trichuris suis* and *Metastrongylus* spp. for piglets (blue), fatteners (black) and adults (red). Scatter plots show values for individual farms **A** and per animal **B** and the means since median and quartile 3 were zero for most data sets. Farm prevalence and EPG values were compared using the Kruskal-Wallis followed by Dunn’s post hoc test (GraphPad Prism). *, *p* < 0.05; **, *p* < 0.01; ***, *p* < 0.001

The frequency of strongylid nematodes was highest in adults (44%), followed by piglets and fatteners (both 28%). Fatteners had the highest frequency of *A. suum* (37%) and *T. suis* (2%). *Metastrongylus* spp. eggs were only detected in one farm (farm 14), where only piglets were tested, resulting in a frequency of 6%. For the frequency calculations of lungworms, only animals were considered, which had access to pasture since their last treatment and therefore could potentially be infected with lungworms (n = 108). The prevalence on farm level and the faecal egg count results of piglets, fatteners and adults are shown in Fig. 2.

### Risk factor analyses

The full data obtained by the questionnaire, as well as the results of the infection rate analyses and the egg shedding intensity analyses via GLMMs are provided in the supplementary data (Additional File 2: Tables S2, and Additional File 3: Tables S3 and S4). The parameters included in the statistical analyses are detailed in Table 3.

**Table 3:** Description of the parameters used for statistical analysis sorted by alphabet

Parameters	Levels	Comments
Anthelmintic class	BZ, ML, none, NA	Class of anthelmintic used at latest treatment
Age group	Piglets, fatteners, adults	
Animal purchase	Yes, no	
Breed	BB, MB, hybrid	BB: German breed, hybrid: first generation of two purebred pig breeds, MB: everything else.
Disinfection	Yes, no	Yes: PAA, H_2_O_2_, CaO, PW, F Flaming, No: None, FA
Farm system	COA, free range/mixed	Free range/mixed farms are farms where at least a part of the animals had access to pasture
Last treatment	6–12 weeks ago, > 16 weeks ago, Never, Unknown	Time since the last treatment of individual animal
Last treatment (days)	(Metric)	Number of days since the last treatment of individual animal
Litter	SL, DL, none	SL: frequent removal of litter; DL: bedding material is added on top of old litter; none: free range animals.
COA clean out	> 2, 2× and 1× per week, < every 2 weeks, None	Frequency of litter removal in COA; none: free range pigs
Pasture access	Yes, no	Pasture access since last treatment
Total animal count	(Metric)	At day of visit
Treatment	Yes, never	Treatment at farm level per age group

BZ, benzimidazoles; ML, macrocyclic lactones; NA, unknown; BB, Bentheim Black Pied pig; MB, mixed breed; PAA, peracetic acid; H_2_O_2_, hydrogen peroxide; CaO, calcium oxide; PW, pressure washing with 90 °C water; F, flaming; FA, formic acid; SL, shallow litter; DL, deep litter; COA, concrete outdoor area

**Fig. 3:**
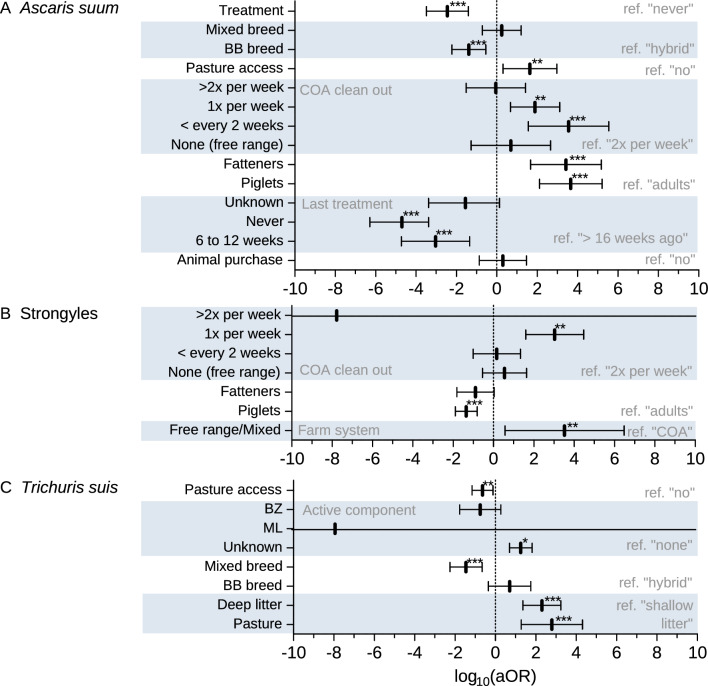
Multivariate generalized linear mixed logistic regression models on the infection status with *Ascaris suum* **A**, strongyles **B** and *Trichuris suis* **C**. The adjusted odds ratios (aOR) with 95% confidence intervals are displayed on a logarithmic scale. *P* values: * *p* ≤ 0.05, ** *p* ≤ 0.01, *** *p* ≤ 0.001

Logistic regression analyses on infection occurrences for *A.* *suum*, strongyles and *T.* *suis* have been calculated in bivariate and multivariate models (Additional file 3: Table S3). The analyses for *A.* *suum* showed that the age group of the animals had a strong influence on the infection status (Fig. 3A). Compared to adults, piglets and fattening pigs had a significantly higher odds of being infected with *A.* *suum* in both the bivariate and multivariate analyses (*p* < 0.001). Animals of the Bentheim Black Pied pig breed (BB) and animals of mixed breed had a significantly lower odds of being infected compared to hybrids (*p* = 0.001) in the bivariate models. With only two farms keeping animals of the BB breed, the protective effect remained in the multivariate analysis (*p* = 0.001). Animals from farms that deworm had significantly fewer ascarid infected animals (*p* < 0.001) than those that never treated their animals with anthelmintics (OR 0.0036). Individuals that received their last deworming 6–12 weeks ago or that had never been treated before had a significantly higher odds (p = 0.013, *p* = 0.012 respectively) of being infected with *A. suum* in the bivariate analysis than animals that had not been dewormed for more than 16 weeks. In contrast, the multivariate analysis showed a significantly lower odds to be positive for ascarids (*p* < 0.001) for both animals that were last dewormed 6–12 weeks ago or animals that had never been treated before. In addition, animals which had access to pasture since the last treatment were infected significantly more often (*p* = 0.015) than animals without access to pasture. Finally, the infection rate for *A.* *suum* increased significantly if the outdoor areas were cleaned out once a week (*p* = 0.002) or less frequently than every 2 weeks (*p* < 0.001) compared to twice a week.

The results of the multivariate analyses for strongyle infections are shown in Fig. 3B. In contrast to *A.* *suum*, for strongyles (bivariate analysis, Additional file 3: Table S3) younger animals had a lower chance of being infected than adult animals and pigs from farms that dewormed had a higher chance of being infected than animals from farms that never dewormed (*p* = 0.003). Additionally, in the multivariate analysis, animals from free range or mixed system farms had a higher risk of being infected with strongyles than those kept in COA farms. As for *A.* *suum*, it was shown that animals showed higher odds of being infected with strongyles if the outdoor run was cleaned out less frequently.

As shown in Fig. 3C, the multivariate analysis for *T.* *suis* revealed that animals with mixed breeds but not the BB breed were significantly less frequently infected compared to hybrid pigs (*p* < 0.001). The results for the anthelmintic class used for the last treatment were inconclusive. Only for animals for which the active component was unknown, mainly fatteners that were last treated by the piglet producer, were found to have a higher chance of being infected with *T.* *suis*. Different to what was expected, the calculations showed a protective effect for animals which had access to pasture compared to animals without access to pasture. Contradictory to this result, free range animals and animals with deep litter outdoor areas had both significantly higher odds to be infected (*p* < 0.001) compared to shallow litter outdoor areas.

### Egg shedding intensity

**Fig. 4:**
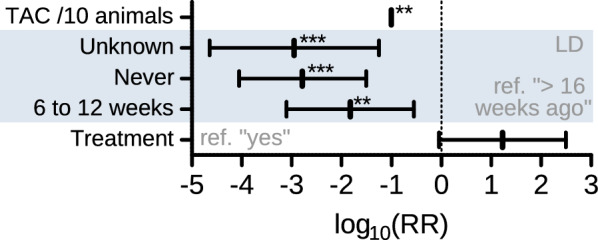
Multivariate generalized linear mixed model of the egg shedding intensity for strongyles. The rate ratios (RR) are displayed on a logarithmic scale with 95% confidence intervals. *P* values: * *p* ≤ 0.05, ** *p* ≤ 0.01, *** *p* ≤ 0.001. TAC/10 animals, RR for total animal count per 10 animals; LD, last deworming

Analyses of egg shedding intensities for strongyles and ascarids were performed and the full results are provided in the Additional file 3: Table S4. Due to low number of positive animals (n = 164), only bivariate analyses were calculated for *A.* *suum* and the results were inconclusive. In contrast, protective factors were identified in a multivariate logistic regression model for strongylid nematodes (Fig. 4), which should be interpreted with caution due to the low number of animals included in the analysis (n = 183). In the multivariate model the following three factors were included: the time since the last treatment, if anthelmintic treatment was used and the total animal count at the day of the visit. The analysis showed that a higher total animal count had a protective effect regarding strongyle egg shedding intensity (*p* = 0.003). Moreover, animals that had been treated 6 to 12 weeks ago had lower egg shedding intensities than animals treated more than 16 weeks ago (*p* = 0.005). But even in animals that never had been treated (*p* < 0.001) or where the last treatment date was unknown (*p* < 0.001), egg excretion was significantly lower than in animals that had not been treated for over 16 weeks.

### Nemabiome analysis

**Fig. 5:**
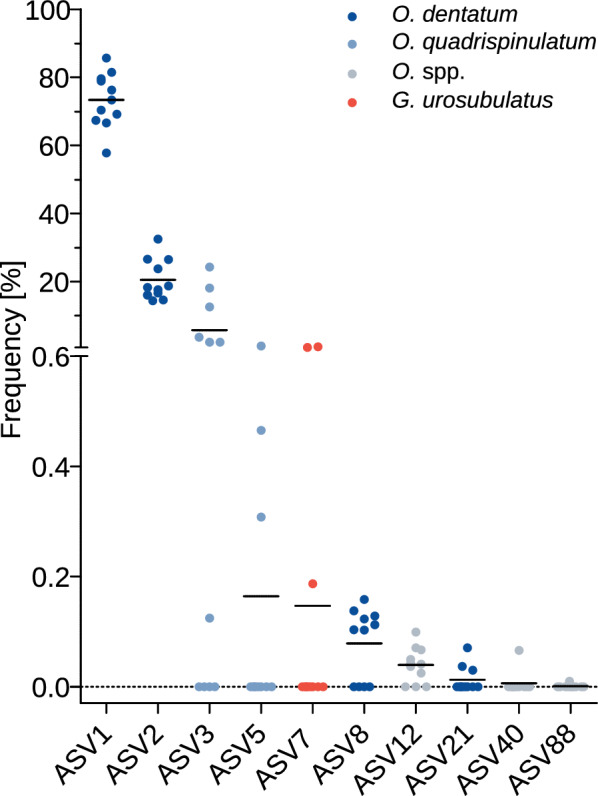
Frequency (%) of amplicon sequence variants (ASVs) per sample. Scatter plots show values for individual samples and the means since median and quartile 3 were zero for most data sets

**Fig. 6:**
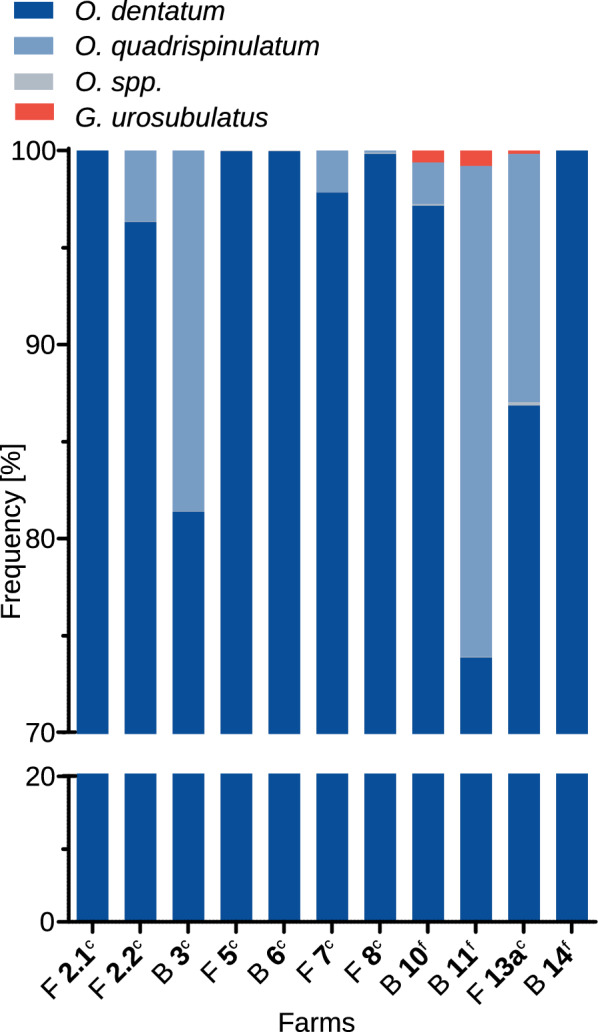
Strongyle species frequencies (%) using deep amplicon sequencing data. The samples are labelled indicating the age groups included in each sample (F, fatteners; B, adults and piglets), the farm (2.1, 3, 5 etc.) and the husbandry form (^f^, free-range; ^c^, concrete outdoor area). Farm 2 was visited twice, in February 2022 (2.1) and November 2022 (2.2)

Deep amplicon sequencing of the ITS-2 region was performed to estimate the nemabiome composition in the studied pig farms. After processing the paired end reads with the dada2 pipeline, between 20,600 and 40,584 reads were generated for each sample (median 28,463). Detailed information about the number of reads remaining after each filtering step are provided in the supplementary data (Additional file 4: Tables S5). In four samples *A. suum* ITS-2 sequences were detected (58–929 reads). An amplification of *A. suum* ITS-2 sequences with NC1/NC2 was shown using DNA from adult worms (Additional file 5: Graphic S6). In one sample (farm 5) four different ASVs with 34 to 264 reads and a total of 1.8% of the reads could be identified as the nematode *Haemonchus contortus*. *Haemonchus contortus* has to the knowledge of the authors not been described from pigs before, Moreover, these pigs did not have access to pastures that might have been contaminated by sheep and goats or wild ruminants such as roe deer. Therefore, there is a high likelihood that these reeds resulted from a laboratory contamination since PCRs for deep amplicon sequencing of goat samples were performed around the same time in the laboratory. Hence, these reads were was not considered to represent a natural infection of pigs with *H. contortus*. With only 10 reads the lungworm infection in farm 14 could be identified as a *M.* *pudendotectus* infection but this does not exclude mixed infections with other species. The reads from *A.* *suum*, *H.* *contortus*, *M.* *pudentotectus* and ASVs that could not be assigned to a nematode species were excluded from further calculations (0–4.4%, median 0.25%), resulting in comparable total read counts per sample, between 20,600 and 40,584 strongyle nematode reads (median 27,206). The number of reads identified as strongyle nematode species were used for parasite frequencies calculations in the different samples. The frequency of each ASV per sample and the assigned species are shown in Fig. 5. The composition of strongyle parasite communities on the 10 different farms and in the 11 different samples (two samples from different visits on farm 2 could be analysed) is shown in Fig. 6.

The deep amplicon sequencing data revealed 10 different ASVs that could be identified as porcine parasites (Fig. 5), of which 4 ASVs could be assigned to the most frequent species *O.* *dentatum*. The most common of these, ASV1 and ASV2 were present in all 11 samples with a mean frequency of 73.3% and 20.5%, respectively. The species *O.* *quadrispinulatum* was represented by the less frequent ASV3 (mean 5.7%) and ASV5 (mean 0.2%). The ASV12 could only be assigned on genus level (*Oesophagostomum*) by IdTaxa and was present in nine samples with a low mean frequency of 0.04%. The sequences of ASV40 and ASV88 were also assigned to the genus *Oesophagostomum* but via BLASTN searches against the GenBank. ASV40 and ASV88 were only found on one farm, respectively. For ASV40 on farm 13a a read count of 18 and a farm frequency of 0.07% was found. ASV88 was found on farm 10, with an even lower read count of three and a farm frequency of 0.01%. When aligned ASV40 and ASV88 are identical but ASV88 is 12 bases longer due to an internal insertion and therefore assigned to another ASV. There was only one *Globocephalus urosubulatus* sequence variant (ASV7), which was present on three different farms, whereof two farms had access to pasture (farm 10, 11) but one farm (farm 13a) had only COAs. There was no detection of *Hyostrongylus rubidus*.

**Fig. 7:**
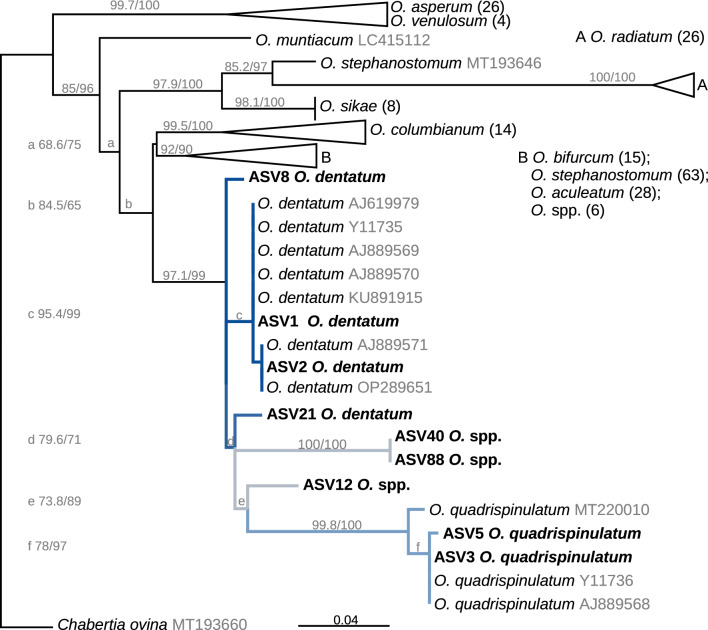
Maximum likelihood phylogenetic tree calculated from ITS-2 sequences of *Oesophagostomum* spp. Sequences of the present study are shown in bold and the amplicon sequence variant (ASV) number is displayed. The sheep parasite *Chabertia ovina* was chosen as outgroup to root the tree. The numbers in brackets after a species indicate the number of sequences hidden behind collapsed phylogenetic tree branches. Branch support is indicated as Shimodaira-Hasegawa-like approximate likelihood ratio test (SH-aLRT) support (%)/ultrafast bootstrap support. Coloured branches represent sequences of porcine *Oesophagostomum* sequences, *O.* *dentatum* (dark blue), *O.* *quadrispinulatum* (light blue) and *Oesophagostomum* spp. (grey)

The *Oesophagostomum* spp. ASVs were analysed together with the available GenBank *Oesophagostomum* spp. ITS-2 sequence data, resulting in the phylogenetic tree shown in Fig. 7. In this tree, the porcine *Oesophagostomum* spp. are clearly separated from those of all other host species. While the *O.* *quadrispinulatum* sequences formed a highly supported monophyletic cluster, this was not the case for *O.* *dentatum* sequences since the *O.* *quadrispinulatum* cluster was placed within the *O.* *dentatum* sequences. Remarkably, there were three ASVs that were not clearly assigned to one or the other of the two species, i.e. the ASVs not assigned to the species level using IdTaxa ASV40/88 and ASV12. They are placed with quite long branches between both groups (Fig. 7).

Overall, there was a low number of observed species with a maximum of three different observed species per sample (Fig. 6). *Oesophagostomum dentatum* occurred in the 11 samples with a median frequency of 97.8% (mean 93.9%). The second most frequently found species was *O.* *quadrispinulatum*, which was detected on 7/11 samples with a significantly lower median frequency of 2.2% (Kruskal-Wallis followed by Dunns’s post hoc test, p < 0.01; mean 5.9%) and with a maximum frequency of 25.3% on farm 11. Other *Oesophagostomum* spp. occurred in eight samples with a median frequency of 0.04% (mean 0.05%). In three samples the hookworm *G.* *urosubulatus* was detected with a median frequency of 0% (mean 0.1%).

## Discussion

In the context of a growing market for meat produced using management systems that allow the animals more natural environments than many industrial production systems, pigs are increasingly kept with access to outside areas ranging from concrete pens to pasture. It is obvious that pigs under such conditions have a different risk to be infected with parasites and it is important to gain a comprehensive understanding of parasite communities and their distribution. In this context it is crucial to raise awareness of alternative parasite control mechanisms and management factors that are able to limit the parasite burden of pigs with outdoor access. Therefore, the aim of the current study was to assess the occurrence of porcine gastrointestinal nematodes and lungworms in German alternatively farmed pigs. Another objective was to specify the porcine intestinal nemabiome via next-generation sequencing on the tested farms. Furthermore, risk factors on farm level for nematode occurrences and egg shedding intensities were investigated.

The results of the present study show, that lungworms, whipworms, ascarids and strongyles are present in German outdoor-reared pigs, but frequency and intensity vary between age groups. The fact that *Strongyloides ransomi* was not detected is consistent with Carstensen, et al. [3] and may be explained by the age of the sampled piglets (about 10 weeks old). Due to the life cycle and rapid development of immunity, *S.* *ransomi* is mainly detected in young suckling piglets [7, 24, 64–68]. However, Roepstorff [67] showed that the same herds of piglets tested positive for *S. ransomi* at 5–8 weeks of age, were also tested positive at the age of 9–12 weeks. This implies that herds positive for *S.* *ransomi* should also be detected by sampling piglets at the age of 10 weeks, as conducted in the present study. Interestingly, previous studies showed that *S.* *ransomi* infections were not as commonly found in northern European organic and conventional pig farms as they used to be [3, 6, 68–70]. Thus, a general decline of prevalence could also be the reason for the lack of detection in the current study. In contrast, Kochanowski et al. [7] found 33.3% of farms with paddocks and 9.4% of farms without paddocks in Poland to be positive for *S.* *ransomi*. On individual animal level, 2% of the samples were positive for *S.* *ransomi*, with the highest prevalence in suckling piglets.

*Metastrongylus* spp. infections are common in wild boars [71–77]. In contrast, previous studies investigating nematodes in organic domestic pigs, infections with lungworms were not common [3, 6, 7, 21, 22, 70]. On the other hand, Wallgren and Pettersson [78] suggested, that *Metastrongylus* spp. infections may be underdiagnosed because of low egg outputs and that they are more common than expected. In the current study lungworm eggs were found on one farm with a very low prevalence and egg counts (6%, 0–5 EPG, respectively). On this particular farm only one age group, piglets, was tested, which therefore does not allow any statement about the age distribution of lungworm infections. Additionally, it should be taken into account that only four farms with access to pasture and consequently access to the intermediate host, various earthworms, were included in this study. In future studies a faecal egg count method with low minimum egg count detection levels (e.g. Mini-FLOTAC, FLOTAC), as used in this study, could potentially prevent underdiagnosing lungworm infections in pigs.

The three most common nematodes found in this study were in increasing frequency *T.* *suis* (5%), *A.* *suum* (27%) and strongyles (32%). However, *A.* *suum* (82%) was present on more of the participating farms than strongyles (70%). Comparable to our results, previous studies investigating farms with outdoor access [3, 6, 7, 21, 22] found with a median of 88% (33.3–100%), 58% (25–100%) and 25% (23–44%) of the farms positive for *A.* *suum*, strongyles and *T.* *suis*, respectively. As expected, *A.* *suum* and *T.* *suis* were most common in fatteners and strongyles in adults [3, 7, 22, 70, 79].

When examining strongyle eggs in pigs in Europe the species *O.* *dentatum* and *O.* *quadrispinulatum* are most commonly expected [6, 70, 80–82]. But also other porcine nematodes, such as *H. rubidus* or hookworms shed strongyle eggs. Traditionally a faecal culture with larval morphology analysis is used to confirm species identity. However, species identification with next-generation sequencing methods is more efficient and offers better chances to detect rare species present with low intensity of egg shedding. While for ruminant nematodes introduced almost a decade ago by Avramenko et al. [33], the use of next-generation deep-sequencing approaches, based on differences in the ITS-2 rDNA locus, has not been described for porcine parasite communities until this study.

Ideally, species-specific biases should be taken into account when quantitatively analysing the deep-sequencing data of mixed parasite communities. However, due to the lack of pure mono-specific populations it was not possible to estimate potential species-specific biases, such as differences in PCR efficiencies or copy numbers of the ITS-2 gene region. Therefore, the computed species frequencies in this study were not corrected for potential species-specific biases.

The analysis of the farm samples revealed three different strongyle species, the by far most common being *O.* *dentatum*, followed by *O.* *quadrispinulatum* and the hookworm *G.* *urosubulatus*. Three ASVs with low read counts could only be assigned to the genus *Oesophagostomum* and the phylogenetic analyses revealed a considerable difference to the *O.* *dentatum* and *O.* *quadrispinulatum* sequences. However, the ITS-2 rDNA locus with a length of approximately 270 bp is not sufficient to determine whether it is actually a separate species.

This is the first report of *G.* *urosubulatus* in domestic pigs in Europe. While hookworm infections are common in wild boars (*Sus scrofa*) in Germany and countries worldwide [71, 72, 75, 76, 83–87] with prevalences up to 95.6% in Germany [72], there are distinctly less reports in domestic pigs (*Sus scrofa domestica*). Two reports for occurrences in domestic pigs from Burkina Faso [88] and China [89] with 10% and 6.7% prevalence respectively, distinguished the *Globocephalus* eggs from strongyle eggs based on the egg size. However, due to high morphological similarities of eggs within the Ancylostomatidae [85], it is questionable if reliable distinction is always possible. Permin et al. [90] were able to detect 2.5% prevalence in faecal examinations and 20% prevalence during necropsy in the Upper East Region of Ghana and Yadav et al. [91] found 8.2% of domestic pigs positive in necropsies in India.

The lungworm infection on farm 14 was associated with the species *Metastrongylus confusus* by deep amplicon sequencing*.* However, the samples contained only very low egg counts and the egg isolation protocol was not optimized to isolate lungworm eggs. Therefore, it has to be assumed that a considerable number of eggs were lost during the egg isolation from the faeces and mixed infections with several *Metastrongylus* species cannot be excluded.

There was no detection of *H.* *rubidus* in the participating farms. The only two previous studies including similar farms as the current study (Northern Europe, alternative pig farms) where not able to find *H.* *rubidus* in domestic pigs [3, 6].

The primers NC1/NC2 are commonly used to amplify the ITS-2 gene region of Clade V nematodes and cestode helminths [33, 38, 44]. Surprisingly, in the current study the NC1/NC2 amplified the ITS-2 rDNA locus of *A.* *suum* in the deep amplicon sequencing. This was confirmed via PCR with extracted DNA from adult *A.* *suum*. However, there was no correlation between farms with high egg counts and farms with high reads of *A.* *suum*. This may be due to the fact, that the egg isolation process was designed to exclude *A.* *suum* eggs and that they only got accidentally mixed with the strongylid eggs. Consequently, it was not possible to quantify the occurrence of *A.* *suum* in the samples using deep amplicon sequencing and it was decided to treat the obtained reads as contamination.

Overall, the metabarcoding based on the ITS-2 gene region is a suitable tool to describe porcine nematode populations. In the future, further analyses will be necessary to estimate species-specific biases in order to quantify reliably species occurrences.

In addition, an aim of this study was to assess risk factors for parasite occurrence and egg shedding intensities for *A.* *suum*, strongyles and *T.* *suis*. Risk factor analysis was performed using generalized linear mixed models [63] to account for farm-level clustering of the samples. It is known that in pigs there is the possibility of false-positive faecal *A.* *suum* or *T.* *suis* egg counts due to passaging after oral uptake of non-infective eggs [92, 93]. However, due to the exclusive use of faecal egg counts it was not possible to assess the actual percentage of false-positive animals in the current study and therefore, for the parasite occurrence model calculations it was assumed that animals with an EPG ≥ 1 were infected.

The discussion in the following section will focus on the multivariate analyses if not indicated otherwise. As one would expect, our results confirmed that the age group of the sampled animals had significant effects for *A.* *suum* and strongyle infections. Younger animals had higher odds (OR 2665–4612) for *A.* *suum* and for strongyles lower odds (OR 0.045–0.13) of being infected. Additionally, in the bivariate analyses, piglets had significantly lower egg shedding intensities compared to adults. This may be due to a poor protective immune response to strongyle infections, leading to persistent infections even in adults [6, 8, 26, 79, 94].

For *A.* *suum* and *T.* *suis,* but not for strongyles, there was a significant effect of the breed on parasite occurrence. This is worth noting, particularly because Roepstorff et al. [8] suggested that breeding for resistant pigs may be a possibility for future sustainable control measures for *A.* *suum* and *T.* *suis*. However, the results of the present calculations are based on only a few farms and therefore further studies with a larger farm numbers per breed would be necessary to corroborate the results.

In the present study, the odds of pigs being infected with *A.* *suum* were lower (OR 0.0036) on farms where anthelmintics were used as could be expected. In contrast, a previous study by Pettersson et al. [23] conducted in Swedish conventional herds, did not find significant effects for the use of anthelmintic drugs. Also, other studies were not able to correlate anthelmintic treatment with nematode prevalences [23–26]. The results for the effect of time elapsed since the last treatment in the bivariate and multivariate analyses for *A.* *suum* were contradictory. There was no observation of effects of treatment on parasite occurrence for strongyles or *T.* *suis*. Generally, the fact that infective nematode eggs and larvae remaining in the environment of the animals may lead to continuous reinfections after each treatment [5, 95] probably complicates the assessment of anthelmintic treatments on nematode prevalences.

However, for the egg shedding intensity for strongyles, a significant higher rate ratio of being infected was observed for those animals which have been treated over 16 weeks ago (only adults), compared to animals treated 6–12 weeks ago (RR 0.015), treated never (RR 0.0016) or last treatment unknown (RR 0.0011). The groups that were never treated or where the date of the last treatment was unknown were mostly piglets and fatteners and therefore, the age distribution may be the reason for the lower egg shedding in these groups.

Previous studies showed a higher risk for parasite infections in organic farms compared to conventional farms [3, 5]. Especially access to pasture with the potential of accumulation of *A. suum* and *T. suis* eggs in the soil [21, 96] poses a risk for infections. Additionally, due to the climate change and the lack of persistent freezing temperatures, the L3 of *Oesophagostomum* spp. may survive up to 8–10 months on pasture [79]. Opposed to that, based on their results Eijck and Borgsteede [6] deduced that access to pasture had a minor role in the epidemiology of *A.* *suum*, strongyles and *T.* *suis.* Our results associated pasture access with higher odds of occurrences for *A.* *suum* and strongyles. However, for *T.* *suis* our results were contradictory. While in the bivariate analysis pasture access resulted in higher odds of being infected, in the multivariate analysis the opposite was observed. This may be due to the small number of positive *T.* *suis* animals and clustering effects. Interestingly, it was shown that free range animals had higher odds being infected with *T.* *suis* than animals with access to COA with shallow litter. This implies, that access to pasture may play a role in the epidemiology of *T.* *suis*.

In the present study animals on deep litter had higher odds being infected with *T.* *suis* compared to shallow litter. The same could be shown for deep litter and free range compared to shallow litter in the bivariate analysis of strongyles. This effect was not shown for *A. suum.* Hence, our results confirm the conclusion of Katakam et al. [96] that deep litter does not pose a significantly higher risk for *A.* *suum* infections compared to shallow litter. However, by implication a deep litter system strongly affects the clean out frequency.

Infrequent cleaning is associated with higher risks of parasite infections [26]. A COA clean out frequency of two times per week or once a week were most common in the participating farms. For *A.* *suum* and strongyles a clean out twice per week was associated with reduced odds of being infected, compared to just once a week. Additionally, in farms performing the clean out more than twice per week no animals tested positive for strongyles or *T.* *suis*. Overall, the results suggest that for all three parasites a COA clean out frequency of at least two times per week can serve as important and powerful control measure.

Several other factors potentially affecting parasite occurrence or egg shedding intensity were investigated, but showed no statistically significant effects. For example, calculations were performed for the last active anthelmintic component used, the application of disinfectants and the purchase of animals. The effects of the All-In-All-Out pig production system could not be assessed, because only one fattening farm (F16) consistently followed the All-In-All-Out system. However, it has to be noted that at this particular farm all samples were tested negative.

## Conclusion

The study demonstrated that *Metastrongylus* spp., *T. suis*, *A.* *suum* and strongyle infections were present in increasing frequencies on German pig farms with outdoor access for the animals. This was the first time that deep amplicon sequencing was used to characterise the nemabiome of pigs. The analysis of the nemabiome showed that the strongyle communities were mainly composed of the nodular worms *O.* *dentatum* and *O.* *quadrispinulatum*. Additionally, it was possible to detect the hookworm *G.* *urosubulatus*. This was the first time *G.* *urosubulatus* was detected in domestic pigs in Europe. Hence, we state that the deep amplicon sequencing based on the ITS-2 is a suitable and powerful tool to distinguish porcine nematodes and to detect rare species. The risk factor analyses demonstrated that treatment reduced the odds for being infected with *A.* *suum* but this effect was not observed for *T.* *suis* or strongyles. In addition, access to pasture and deep litter were associated with higher odds of being infected. A frequent clean out of the outdoor areas decreased the risk of being infected with *A. suum* and strongyles. Furthermore, a potential genetic component with some breeds being less frequently infected with *A.* *suum* and *T.* *suis* than others. Overall, to confirm the presented results it would be necessary to conduct similar studies with a larger study population.

## Supplementary Information

Supplementary Material 1: Translated questionnaire.

Supplementary Material 2: Farm and egg count data.

Supplementary Material 3: Results of the bivariate and multivariate risk factor analyses for Ascaris suum, strongyles and Trichuris suis. Table S3 reports results of the logistic regression analysis on infection status and Table S4 of the negative binomial regression analysis on egg counts.

Supplementary Material 4: Filtering statistics of the deep amplicon sequencing reads.

Supplementary Material 5: Amplification of Ascaris suum genomic DNA with primers NC1/NC2.

## Data Availability

The dataset supporting the conclusions of this article is included within the article (and its additional files 1-5). Raw deep sequencing data were deposited in the short read archive (SRA) of GenBank under the BioProject ID PRJNA1111032 with the BioSample IDs SAMN41380801–SAMN41380811. The ITS-2 sequence data for sequence variants of *Oesophagostoumum* spp. that were not in GenBank before, were deposited in GenBank under the accession no. PP785332–PP785340.
