# Supplementary material for: Gastrointestinal nematodes in German outdoor-reared pigs based on faecal egg count and next-generation sequencing nemabiome data
Source: Porcine Health Manag. 2024 Sep 12;10:33. doi: 10.1186/s40813-024-00384-8 (PMC11391852; doi:10.1186/s40813-024-00384-8)
Supplement: Supplementary file 1 — Supplementary Material 1: Translated questionnaire. [file 40813_2024_384_MOESM1_ESM.pdf]

Additional file 1: Text S1 – Questionnaire

A. Contact information

Study farm ID: ______

1. Name ____
2. Farm owner ____
3. Address (street, postal code, place) ____
4. Contact information: (email, phone, fax) ____

5. Date of visit ____________

B. Questionnaire

General information on pig husbandry

1. Pig breeds/genetics ____
2. Is your company EU organic certified? ☐ Yes ☐ No
3. Participation „Demeter", „Neuland" etc. ____
4. Animal husbandry form ☐ Free-range ☐ Concrete outdoor area
5. Total animal count today ______ annual average _______
    a. of which sows (today) _______ Sows
    b. of which boars (today) _______ Boars
    c. of which suckling piglets (today) _______ Suckling piglets (until weaning)
    d. of which weaners (today) _______ Weaners
    e. of which fatteners (today) _______ Fatteners
6. Age of weaning ____________________________
7. Age of relocation in fattening units ____________________________
8. Farm system ☐ Breeding ☐ Fattening ☐ _______________
9. Occupation ☐ Main occupation ☐ Secondary occ. ☐ Hobby
10. How many pigs are purchased per year? No. of pigs _______
    And from which regions? ☐ ____
11. Do you separate purchased animals before stabling them? For how long? ☐ Always. ☐ Most of the time. ☐ Seldom. ☐ Never. Quarantine time ______________

12. Do you deworm purchased animals before they are given access to the pasture or before stabling? ☐ Always. ☐ Most of the time. ☐ Seldom. ☐ Never. ☐ I only buy animals that have been dewormed beforehand.
13. Do you examine animals coproscopically before stabling? ☐ Always. ☐ Most of the time. ☐ Seldom. ☐ Never.
14. Own or purchased feed ☐ Own feed ☐ Purchased feed
15. Producer purchased feed ______________________________
16. Which components are fed? ______________________________

<u>General information on the form of husbandry</u>

**Form of husbandry**<br>If applicable, please indicate the size of the total outdoor area in m^2 or ha. If not applicable, please cross out the relevant field.			**Specifications for outdoor areas**<br>(please check off where applicable, multiple answers possible; please indicate solid floor areas as a percentage of the total outdoor area)					
Total outdoor area								
Dry sows								
Lactating sows								
Weaners (ca. 7.-10. weeks old)								
Fatteners								

*______________________

17. Condition of the paved surfaces with regard to cleaning & disinfection ☐ Good ☐ Moderate ☐ Poor (porous/cracked)

Free range

18. Are the pigs being stabled in winter? ☐ Yes ☐ No
19. Time of stabling in winter? ______________________
20. How are the pigs watered in the pasture? ☐ Drinking trough ☐ Nipple drinkers ☐ Access to natural water sources

Hygiene management

21. All-In-All-Out system ☐ Yes ☐ No

    Relocation of huts on pasture ☐ Yes ☐ No

22. Pasture management ☐ Continous grazing ☐ Rotational grazing ☐ Strip grazing ☐ others ________

23. Pasture hygiene: if yes, how and how often? ☐ Yes________________________________ ☐No

24. How often do you muck out the runs? ☐ ≥ 3x per week (pw) ☐ 2x pw ☐ 1x pw. ☐ every two weeks ☐ less frequent

25. How often is basic cleaning carried out? ____

26. Which disinfectant do you use? __________

27. How do you disinfect? (exposure time, method, temperature) ____

28. How often do you use disinfectant after cleaning? ☐ Always. ☐ Most of the time. ☐ Seldom. ☐ Never.

Treatment/Deworming

29. Do you follow a regular deworming schedule? ☐ Always. ☐ Most of the time. ☐ Seldom. ☐ Never.

30. Which age groups are dewormed how often and when?

    a. Suckling piglets ____________________________

    b. Weaners ____________________________

    c. Fatteners ____________________________

    d. Sows ____________________________

    e. Boars ____________________________

31. Are all animals in the group living together dewormed at the same time? ☐ Yes ☐ No

32. What criteria do you use to decide on deworming?

    ☐ Slaughter results

    ☐ Coproscopical examinations

    ☐ Deworming schedule created by _____________

    ☐ Recommendations from _____________________

    ☐ Health condition of animals

    ☐ ____

33. Which form of application do you prefer (i.e. is most frequently used)?	☐ Feed ☐ Drinking water ☐ Injection<br>☐ Others ______________________
34. If applied via the feed: Who mixes the medication into the feed?	☐ Farmer/employee ☐ Feed producer<br>☐ ____
35. How is it mixed in? For how long?	____
36. How is the weight of the animals determined for the dosage?	☐ Scale ☐ Estimation<br>☐ Others ________________________________
37. Have you used any of the following medications to treat nematodes in the last year?	
a. **Ivermectin** (Ivomec-S®, Alfamectin®) Form of application	a. ☐ Product ______________________________<br>☐ Feed ☐ Drinking water ☐ Injection
b. **Doramectin** (Dectomax S®) Form of application	b. ☐ Product ______________________________<br>☐ Feed ☐ Drinking water ☐ Injection
c. **Fenbendazole** (Panacur®, Pigfen®, Fenbendatat®) Form of application	c. ☐ Product ______________________________<br>☐ Feed ☐ Drinking water ☐ Injection
d. **Flubendazole** (Flimabend®, Flubenol®, Frommex®, Flimabo®, u.a.) Form of application	d. ☐ Product ______________________________<br>☐ Feed ☐ Drinking water ☐ Injection
e. **Levamisole** (Concurat-L®) Form of application	e. ☐ Product ______________________________<br>☐ Feed ☐ Drinking water ☐ Injection
f. **Others** Form of application	f. ☐ Product ______________________________<br>☐ Feed ☐ Drinking water ☐ Injection
38. Duration of treatment (days)?	Weaners ______________________________<br>Fatteners______________________________<br>Sows__________________________________
39. Do you change the product regularly?	☐ Several times a year ☐ Once a year ☐ Every couple of years ☐ Per production cycle<br>☐ Several times per production cycle ☐ No.
40. Is the success of the treatment monitored by testing fecal samples?	☐ Always. ☐ Most of the time. ☐ Seldom.<br>☐ Never.
41. If no, reasons why not?	____

42. Have you already had problems with subjectively ineffective deworming products? ☐ Yes ☐ No
    a. If yes, which product? ______________________
    b. Follow up examination by a veterinarian? ☐ Yes ☐ No
43. Who plans the treatment? ☐ Farm owner ☐ Veterinarian ☐ Animal health service

44. Do you feel sufficiently well advised by your vet/animal health service regarding deworming strategies? ☐ Yes ☐ Rather yes ☐ Rather no ☐ No
45. Do you feel well informed about deworming? ☐ Yes ☐ Rather yes ☐ Rather no ☐ No
46. Would you like more information about deworming? ☐ Yes ☐ Rather yes ☐ Rather no ☐ No
47. Do you think regular faecal examinations on your farm make sense? ☐ Yes ☐ Rather yes ☐ Rather no ☐ No

Deworming managment

48. Do you think it makes sense to deworm all animals in a herd?
    ☐ Yes ☐ Rather yes ☐ Rather no ☐ No
49. Do you change the pasture or pen after deworming? ☐ Yes ☐ Rather yes ☐ Rather no ☐ No
50. How important do you think basic cleaning is for worm control? ☐ Important ☐ Less important ☐ Not important ☐ No relevance
51. How important do you think pasture management is for worm control? ☐ Important ☐ Less important ☐ Not important ☐ No relevance
52. Do you carry out basic cleaning after deworming? ☐ Yes ☐ Rather yes ☐ Rather no ☐ No
53. Alternatives to anthelminthics - what are you aware of? ☐ Feed (tannins, fungi, etc.) ☐ Breeding ☐ Pasture Management ☐ Others:______________________________
54. Have you already had experience with one of the alternatives mentioned above? If so, with what? ☐ No ☐ Yes: ______________________

Diseases

1. How high do you rate the occurrence of the following disease complexes in your company? Scale from 1-10: from 1 no occurrence in my company to 10 high occurrence.
   - Airway diseases ______
   - Poorley developed piglets _______
   - Diarrhea ________
     In particular, which age group?
     ☐ Suckling pigets ☐ Weaners
     ☐ Fattener ☐ Sows
   - Abortions/still birth ________
   - Condemned livers _______
2. Do you have a sow planner or fattening planner? Or something similar?
   ☐ Sow planner ☐ Fattening planner
   ☐ ____
3. Reasons for animal losses? ____
4. Proportion of piglet losses due to diarrhea: ☐ 0-10 % ☐ 11-20% ☐ 21-30% ☐ 31-40% ☐ 41-50% ☐ 51-60% ☐ 61-70% ☐ 71-80% ☐ 81-90% ☐ 91-100%
5. Isolation of sick animals? ☐ Always ☐ If possible ☐ Seldom ☐ Never
6. Where are they isolated? ____
