# Supplementary material for: Gastrointestinal nematodes in German outdoor-reared pigs based on faecal egg count and next-generation sequencing nemabiome data
Source: Porcine Health Manag. 2024 Sep 12;10:33. doi: 10.1186/s40813-024-00384-8 (PMC11391852; doi:10.1186/s40813-024-00384-8)
Supplement: Supplementary file 3 — Supplementary Material 3: Results of the bivariate and multivariate risk factor analyses for Ascaris suum, strongyles and Trichuris suis. Table S3 reports results of the logistic regression analysis on infection status and Table S4 of the negative binomial regression analysis on egg counts. [file 40813_2024_384_MOESM3_ESM.pdf]

Additional file 3: Table S3 – Statistical analysis: Infection status *Ascaris suum*

**Infection status**	***N***	***Actual % infected***	***95% CI***		***N*$_{farm}$**	**Bivariable**				**Multivariable**			
						OR	***95% CI***		***P***	***aOR***	***95% CI***		***P***
Ascaris suum													
Total	607	27.0	23.6	30.6	17								
Age group													
Piglets	217	23.0	17.9	29.1	10	6.84	2.40	19.55	**<0.001**	4612.38	131.24	1.62x10^{5}	**<0.001**
Fatteners	296	36.5	31.2	42.1	10	16.27	4.52	58.10	**<0.001**	2665.67	46.99	1.51x10^{5}	**<0.001**
Adults	94	6.4	3.0	13.2	9	1				1			
Breed													
Mixed breed	262	21.8	17.2	27.1	10	0.110	0.029	0.417	**0.001**	1.75	0.192	16.04	0.618
Bunte Bentheimer	78	34.6	25.0	45.7	2	0.070	0.015	0.322	**<0.001**	0.041	0.0059	0.287	**0.001**
Hybrid	267	30.0	24.8	35.7	8	1				1			
Last treatment													
6-12 weeks ago	166	12.6	8.4	18.6	7	7.70	1.52	38.78	**0.013**	9.24x10^{-4}	1.90x10^{-5}	0.045	**<0.001**
> 16 weeks ago	37	10.8	4.3	24.7	4	1				1			
Never	325	28.9	24.3	34.1	12	5.57	1.45	21.39	**0.012**	3.86x10^{-5}	5.15x10^{-7}	0.0029	**<0.001**
Unknown	79	60.0	46.0	67.3	3	50.34	3.04	834.55	**0.006**	0.024	4.15x10^{-4}	1.36	0.069
Last treatment (days)	203	12.3	8.5	17.5	10	1.02	1.00	1.04	**0.028**				
Active component													
None	325	28.9	24.3	34.1	12	1							
BZ	205	18.0	13.4	23.9	9	2.56	1.01	6.46	**0.047**				
ML	28	0	0	12.1	2	5.08x10^{-10}	0	Inf	0.997				
NA	49	67.3	53.4	78.8	2	16.45	0.62	435.37	0.093				
Treatment													
Yes	440	26.6	22.7	30.9	15	0.471	0.172	1.289	0.143	0.0036	3.29x10^{-4}	0.039	**<0.001**
Never	167	28.1	21.9	35.4	7	1				1			
Farm system													
Concrete outdoor area	457	31.5	27.4	35.9	13	1							
Free range/Mixed	150	13.3	8.8	19.7	4	0.68	0.064	7.27	0.750				
Pasture access since last treatment													
No	499	25.1	21.4	29.0	16	1				1			
Yes	108	36.1	27.7	45.5	5	3.75	1.45	9.65	**0.006**	43.83	2.06	933.32	**0.015**
COA clean out													
> 2x per week	59	20.3	12.0	32.3	2	2.15	0.127	36.38	0.597	0.90	0.031	26.44	0.951
2x per week	241	7.0	4.5	11.0	7	1				1			
1x per week	195	44.6	37.8	51.6	6	17.31	2.54	117.84	**0.004**	78.60	4.78	1291.87	**0.002**
< every two weeks	56	67.9	54.8	78.6	2	45.69	4.61	452.50	**0.001**	3588.89	36.52	3.53x10^{5}	**<0.001**
None (free range)	56	17.6	10.0	29.8	2	7.90	0.913	68.34	0.060	4.96	0.053	462.52	0.489
Litter													
Shallow litter	405	22.0	18.2	26.3	12	1							
Deep litter	146	44.5	36.7	52.6	5	5.95	0.873	40.50	0.068				
None (free range)	56	17.6	10.0	29.8	2	6.81	0.660	70.09	0.107				
Total animal count (/10 animals)	607					0.099	0.099	10.00	0.480				
Disinfection													
Yes	360	36.4	31.6	41.5	11	3.04	0.39	23.46	0.287				
No	247	13.4	9.7	18.1	6	1							
Animal purchase													
Yes	302	42.7	37.3	48.4	9	6.05	0.96	37.99	0.055	2.00	0.136	29.39	0.613
No	305	11.5	8.4	15.5	8	1				1			

Additional file 3: Table S3 – Statistical analysis: Infection status strongyles

Infection status	N	*Actual % infected*	*95% CI*		N_{farm}	**Bivariable**				**Multivariable**			
						OR	*95% CI*		*P*	*aOR*	*95% CI*		*P*
**Gastrointestinal strongyles**													
Total	577	31.7	28.1	35.6	17								
Age group													
Piglets	217	27.6	22.1	34.0	10	0.050	0.015	0.169	**<0.001**	0.045	0.013	0.16	**<0.001**
Fatteners	266	30.8	25.6	36.6	10	0.023	7.7x10^{-4}	0.847	**0.040**	0.13	0.0032	4.99	0.271
Adults	94	43.6	34.0	53.7	9	1				1			
Breed													
Mixed breed	262	31.7	26.3	37.5	10	4.36	0.069	276.72	0.486				
Bunte Bentheimer	58	69.0	56.2	79.4	2	22.27	0.013	3.83x10^{4}	0.414				
Hybrid	257	23.3	18.6	28.9	8	1							
Last treatment													
6-12 weeks ago	136	31.6	24.4	39.8	7	0.17	0.026	1.22	0.079				
> 16 weeks ago	37	54.1	38.4	69.0	4	1							
Never	325	28.3	23.7	33.4	12	0.019	0.0034	0.12	**<0.001**				
Unknown	79	35.4	25.8	46.4	3	0.062	9.11x10^{-6}	28.71	0.279				
Last treatment (days)	174	36.2	29.4	43.6	9	1.020	1.00	1.04	0.055				
Active component													
None	325	28.3	23.7	33.4	12	1							
BZ	175	26.9	20.8	33.9	9	7.59	2.05	28.11	**0.002**				
ML	28	57.1	39.1	73.4	2	231.52	1.56	3.44x10^{4}	**0.033**				
NA	49	57.1	43.3	70.0	2	24.30	0.0081	7.33x10^{4}	0.435				
Treatment													
Yes	410	38.0	33.5	42.8	15	51.83	3.82	704.16	**0.003**				
No	167	16.2	11.4	22.5	7	1							
Farm system													
Concrete outdoor area	427	27.9	23.8	32.3	13	1				1			
Free range/Mixed	150	42.7	35.0	50.7	4	234.6	0.374	1.47x10^{5}	0.096	3342.22	3.75	2.98x10^{6}	**0.019**
Pasture access since last treatment													
No	496	30.5	26.5	34.8	16	1							
Yes	108	37.0	28.5	46.4	5	14.21	2.15	94.08	**0.006**				
COA clean out													
> 2x per week	59	0	0	6.1	2	3.22x10^{-10}	0	Inf	0.998	1.67x10^{-8}	0	Inf	0.998
2x per week	241	22.4	17.6	28.1	7	1				1			
1x per week	165	63.6	56.1	70.6	6	33.48	0.297	3775.81	0.145	2077.59	3.49	3.3x10^{5}	**0.017**
< every two weeks	56	1.8	0.3	9.4	2	16.59	0.1889	1458.99	0.219	1.46	0.014	156.74	0.875
None (free range)	56	41.1	29.2	54.1	2	10.56	0.235	475.17	0.225	3.53	0.045	275.00	0.570
Litter													
Shallow litter	375	40.0	35.2	45.0	12	1							
Deep litter	146	6.8	3.8	12.1	5	85.74	2.45	29995.48	**0.014**				
None (free range)	56	41.1	29.2	54.1	2	215.59	3.12	1.49x10^{4}	**0.013**				
Total animal count (/10 animals)	577					0.0999	0.0996	10.01	0.264				
Disinfection													
Yes	360	25.6	21.3	30.3	11	0.0081	3.3x10^{-5}	2.02	0.087				
No	217	41.9	35.6	48.6	6	1							
Animal purchase													
Yes	272	41.9	36.2	47.8	9	1.24	0.010	149.55	0.929				
No	305	22.6	18.3	27.6	8	1							

Additional file 3: Table S3 – Statistical analysis: Infection status *Trichuris suis*

**Infection status**	**N**	***Actual % infected***	***95% CI***		**N_{farm}**	**Bivariable**				**Multivariable**			
						OR	***95% CI***		***P***	***aOR***	***95% CI***		***P***
Trichuris suis													
Total	607	5.3	3.8	7.3	17								
Age group													
Piglets	217	1.4	0.5	4.0	10	0.12	0.016	0.95	**0.045**				
Fatteners	296	9.5	6.6	13.3	10	1							
Adults	94	1.1	0.2	5.8	9	0.61	0.01	2.03	0.158				
Breed													
Mixed breed	262	1.5	0.6	3.9	10	0.131	0.020	0.866	**0.035**	0.035	0.0056	0.22	**<0.001**
Bunte Bentheimer	78	2.6	0.7	8.9	2	0.91	0.087	9.58	0.940	5.03	0.45	56.75	0.191
Hybrid	267	9.7	6.7	13.9	8	1				1			
Last treatment													
6-12 weeks ago	166	0	0	2.3	7	$3.79x10^{-10}$	0	Inf	0.999				
> 16 weeks ago	37	2.7	0.5	13.8	4	1							
Never	325	8.3	5.8	11.8	12	0.37	0.015	8.95	0.537				
Unknown	79	5.1	2.0	12.3	3	0.24	0.0012	46.69	0.596				
Last treatment (days)	203	0.5	0.09	15.0	10	1.15	NA	NA	NA				
Active component													
None	325	8.3	5.8	11.8	12	1				1			
BZ	205	0.5	0.1	2.7	8	0.16	0.015	1.77	0.136	0.177	0.017	1.83	0.146
ML	28	0	0	12.1	2	$5.23x10^{-8}$	0	Inf	0.999	$1.13x10^{-8}$	0	Inf	0.998
NA	49	8.2	3.2	19.2	2	2.20	0.021	232.10	0.740	17.93	1.91	168.12	**0.011**
Treatment													
Yes	440	4.2	2.8	6.6	15	4.95	0.23	106.30	0.306				
Never	167	7.8	4.6	12.9	7	1							
Farm system													
Concrete outdoor area	457	6.6	4.6	9.2	13	1							
Free range/Mixed	150	1.3	0.4	4.7	4	0.25	0.0031	20.54	0.539				
Pasture access since last treatment													
No	499	4.4	2.9	6.6	16	1				1			
Yes	108	9.3	5.1	16.2	5	6.85	1.04	44.89	**0.045**	0.23	0.069	0.75	**0.015**
COA clean out													
> 2x per week	59	0	0	6.1	2	$3.78x10^{-5}$	0	Inf	0.999				
2x per week	241	0	0	1.6	7	1							
1x per week	195	10.8	7.2	15.9	6	$3.16x10^{9}$	0	Inf	0.998				
< every two weeks	56	16.1	8.7	27.8	2	$5.71x10^{9}$	0	Inf	0.998				
None (free range)	56	3.6	1.0	12.1	2	$1.39x10^{9}$	0	Inf	0.998				
Litter													
Shallow litter	405	1.7	0.8	3.5	12	1				1			
Deep litter	146	15.8	10.7	22.5	5	41.83	1.27	1381.22	**0.036**	200.33	23.30	1722.17	**<0.001**
None (free range)	56	3.6	1.0	12.1	2	19.49	0.15	2566.96	0.233	623.08	19.08	$2.03x10^{4}$	**<0.001**
Total animal count (/10 animals)	607					0.10	0.099	10.03	0.378				
Disinfection													
Yes	360	7.5	5.2	10.7	11	2.41	0.051	113.48	0.654				
No	247	2.0	0.9	4.7	6	1							
Animal purchase													
Yes	302	5.6	3.5	8.8	9	2.96	0.055	158.46	0.593				
No	a305	4.9	3.0	7.9	8	1							

Additional file 3: Table S4 – Statistical analysis: Egg shedding intensity – *A. suum*

**Egg shedding intensity**	***N***	***median***	***IQR***		***N$_{farm}$***	**Bivariable**			
			25%	75%		***RR***	***95% CI***		***P***
Ascaris suum									
Total	164	8	2	387.8	14				
Age group									
Piglets	50	47,5	2	486.3	6	1.43	0.25	8.30	0.689
Fatteners	108	8	2	358	8	1			
Adults	6	34	3.25	85.25	2	0.37	0.015	8.69	0.528
Breed									
Mixed breed	57	23	1	319	8	0.74	0.15	3.67	0.71
Bunte Bentheimer	27	2	1	273	2	1.12	0.078	16.15	0.934
Hybrid	80	10	4	555.3	6	1			
Last deworming									
6-12 weeks ago	21	2	1	7	3	0.48	0.03	7.79	0.609
> 16 weeks ago	4	34	7.25	106.5	2	1			
Never	94	21	2	528	9	8.70	0.62	121.6	0.108
Unknown	45	9	4	497.8	3	20.30	1.33	309.6	**0.030**
Active component									
None	94	19.5	2	541.5	9	1			
BZ	37	3	1	10	5	0.057	0.021	0.155	**<0.001**
NA	33	116	5.5	838.5	2	3.28	0.95	11.34	0.061
ML	0								
Treatment									
Yes	117	21	3	474	10	1			
Never	47	4	2	215	4	0.93	0.11	7.89	0.947
Farm system									
Concrete outdoor area	144	8	2	332.5	10	1			
Free range/Mixed	20	77	8.75	723.3	4	1.98	0.17	23.28	0.588
Pasture access since last treatment									
No	125	5	2	242	12	1			
Yes	39	213	7	900	3	2.34	0.45	12.04	0.310
Outdoor area clean out									
> 2x per week	12	3.5	1	4	1	0.025	0.0012	0.54	**0.018**
2x per week	17	23	2.5	576.5	4	1			
1x per week	87	5	2	242	6	0.23	0.023	2.35	0.216
< every two weeks	38	236.5	7.75	813.5	2	0.688	0.040	11.94	0.797
None (free range)	10	192	20	948.5	2	0.71	0.025	20.20	0.842
Litter									
Shallow litter	89	6	1.5	324	5	1			
Deep litter	65	8	4	474	8	0.43	0.060	3.05	0.396
None (free range)	10	192	20	948.5	2	1.66	0.058	47.71	0.768
Total animal count (/10 animals)	164					0.099	0.099	10.00	0.213
Disinfection									
Yes	131	8	2	466	9	1.36	0.167	11.12	0.773
No	33	26	2	253	5	1			
Animal purchase									
Yes	129	8	2	435	8	1.21	0.15	9.89	0.859
No	35	2	1	34	6	1			

Additional file 3: Table S4 – Statistical analysis: Egg shedding intensity strongyles

Egg shedding intensity	*N*	*median*	*IQR*		N_{farm}	Bivariable				Multivariable			
			25%	75%		*RR*	*95% CI*		*P*	*RR*	*95% CI*		*P*
**Gastrointestinal strongyles**													
Total	183	81	8	281	12								
Age group													
Piglets	60	86.5	6	254.5	6	0.074	0.035	0.16	**<0.001**				
Fatteners	82	28	7.75	104.3	5	0.060	0.0012	3.06	0.161				
Adults	41	661	136	1490	6	1							
Breed													
Mixed breed	83	90	16	376	6	1.25	0.031	59.11	0.877				
Bunte Bentheimer	40	107.5	21	372.3	2	0.57	0.19	1.69	0.310				
Hybrid	60	48	4	245.5	5	1							
Last deworming													
6-12 weeks ago	43	39	10	157	4	0.18	0.047	0.70	**0.013**	0.015	7.91x10^{-4}	0.28	**0.005**
> 16 weeks ago	20	809	295	1431	2	1				1			
Never	92	111.5	25	278.3	8	0.023	0.0085	0.060	**<0.001**	0.0016	8.82x10^{-5}	0.031	**<0.001**
Unknown	28	4.5	3	17.5	2	0.022	1.58x10^{-4}	2.99	0.128	0.0011	2.28x10^{-5}	0.056	**<0.001**
Last treatment (days)	63	108	21	764	6	1.01	0.994	1.03	0.182				
Active component													
None	92	111.5	25	278.3	8	1							
BZ	47	86	21	444	4	10.82	4.23	27.69	**<0.001**				
ML	16	564	9.5	1431	2	32.85	12.24	88.12	**<0.001**				
NA	28	4.5	3	17.5	2	1.09	0.0051	233.24	0.976				
Treatment													
Yes	156	75.5	12.5	246.8	10	1				1			
Never	27	216.0	3	3660	3	0.030	0.011	0.086	**<0.001**	16.71	0.90	309.59	0.059
Farm system													
Concrete outdoor area	119	34	5	157	8	1							
Free range/Mixed	64	191	38.75	1040	4	2.53	0.057	110.88	0.631				
Pasture access since last treatment													
No	143	61	6	216	10	5.29	1.91	14.65	**0.001**				
Yes	40	191	36.5	766.8	4	1							
Outdoor area clean out													
> 2x per week	0												
2x per week	54	111.5	4.75	1377	4	1							
1x per week	105	68	12	183.5	6	1.74	0.044	67.89	0.768				
< every two weeks	1					2.19x10^{-8}	0	Inf	0.995				
None (free range)	23	125	30	281	2	0.11	5.9x10^{-4}	19.94	0.404				
Litter													
Shallow litter	150	68	6.75	256.3	9	1							
Deep litter	10	516	81.25	876.8	3	3.55	0.010	1253.74	0.672				
None (free range)	23	125	30	281	2	0.320	0.0019	52.53	0.661				
Total animal count (/10 animals)	183					0.0998	0.0996	0.0999	**0.016**	0.0998	0.0996	0.0999	**0.003**
Disinfection													
Yes	92	22.5	4	125.5	7	0.25	0.0070	8.70	0.44				
No	91	143	49	528	5	1							
Animal purchase													
Yes	114	48	6.75	167.5	6	5.71	0.13	249.08	0.366				
No	69	161	29	834	6	1							
