# Supplementary material for: Gastrointestinal nematodes in German outdoor-reared pigs based on faecal egg count and next-generation sequencing nemabiome data
Source: Porcine Health Manag. 2024 Sep 12;10:33. doi: 10.1186/s40813-024-00384-8 (PMC11391852; doi:10.1186/s40813-024-00384-8)
Supplement: Supplementary file 4 — Supplementary Material 4: Filtering statistics of the deep amplicon sequencing reads. [file 40813_2024_384_MOESM4_ESM.pdf]

Additional file 4: Table S5 – Reads filtering statistics

Farm ID	raw	filtered	denoised_fwd	denoised_rev	merged	no_chim
2.1	29796	20639	20632	20627	20600	20600
2.2	37950	27222	27215	27219	27110	26842
3	53070	43275	43242	43166	43032	40584
5	40221	30830	30825	30749	30732	30732
6	38768	30092	30086	29984	29980	29980
7	30784	22978	22963	22961	22839	22761
8	30952	24448	24412	24352	24342	24342
10	36414	30052	30037	30052	29931	29792
11	33107	26684	26633	26649	26477	24550
13	37065	29035	28927	28907	28750	28431
14	37309	29858	29847	29754	29712	29712
