# Supplementary material for: Gastrointestinal nematodes in German outdoor-reared pigs based on faecal egg count and next-generation sequencing nemabiome data
Source: Porcine Health Manag. 2024 Sep 12;10:33. doi: 10.1186/s40813-024-00384-8 (PMC11391852; doi:10.1186/s40813-024-00384-8)
Supplement: Supplementary file 5 — Supplementary Material 5: Amplification of Ascaris suum genomic DNA with primers NC1/NC2. [file 40813_2024_384_MOESM5_ESM.pdf]

NC1/NC2 – PCR *Ascaris suum* Additional file 5 - S6

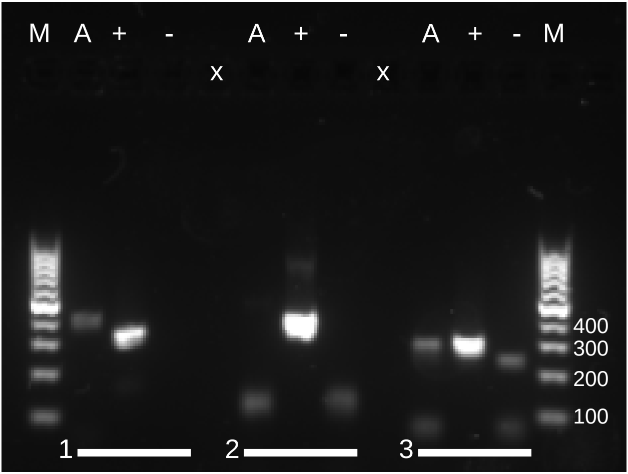

M: GeneRuler 100 bp Ladder
A: *Ascaris suum* DNA
+: Positiv control (*Oesophagostomum dentatum* DNA)
-: Negativ control (PCR-grade water)
x: Empty gel pocket

1: PCR with NC1/NC2 primers
2: PCR with NC1/NC2 primers with Illumina adapters
3: 28S-PCR (control of DNA-Isolation *Ascaris suum*)
